# A Novel Endovascular Treatment for Recurrent Secondary Aorto-Enteric Fistula in a Patient With Prior Infra-Renal Aortic Ligation

**DOI:** 10.7759/cureus.34195

**Published:** 2023-01-25

**Authors:** Calvin Cheong, Rajesh Dharmaraj, Amos Tan, Shao J Ong, Gopinathan Anil

**Affiliations:** 1 Radiology, National University Hospital, Singapore, SGP; 2 Vascular Surgery, National University Hospital, Singapore, SGP

**Keywords:** ct angiogram, aortic diseases, percutaneous endovascular repair, aorto-enetric fistula, endovascular stenting, endovascular procedures

## Abstract

This report highlights the use of novel endovascular techniques in a 68-year-old male patient with massive hematemesis from a recurrent secondary aorto-enteric fistula (SAEF). With a prior history of infrarenal aortic ligation and the location of the SAEF being at the aortic sac, we explain the considerations for the techniques used and how we were able to stop the bleeding using percutaneous transarterial embolotherapy.

## Introduction

Aorto-enteric fistula (AEF) is an abnormal communication between the aorta and the gut. Based on etiology, it could be primary or secondary, with the latter usually following aortic surgery [1]. In secondary aorto-enteric fistula (SAEF), conventional treatment is surgical. However, endovascular options are often reasonable and sometimes may be required as a bridge to surgery [2]. We report a novel endovascular strategy employed in treating a recurrent SAEF in a patient with previous infra-renal aortic ligation and extra-anatomic bypass.

## Case presentation

**Figure 1:**
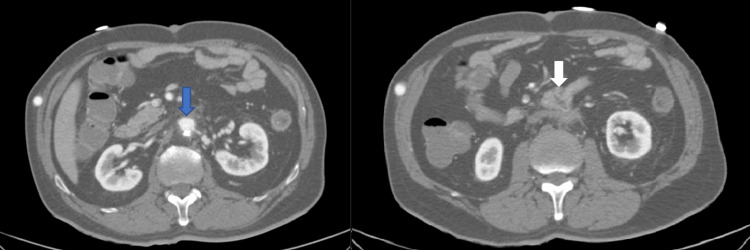
(Left) A remnant infrarenal aortic sac with a 12 mm lumen (blue arrow). (Right) A loop of the bowel (duodenum) is seen apposed to the anteroinferior aspect of the aortic sac (white arrow).

A 68-year-old male presented in shock with hematemesis. His blood pressure at presentation was 87/61 mmHg, and his heart rate was 108 beats per minute. He had a history of infected aortobifemoral bypass complicated with SAEF. Seven years back, it was successfully treated with a right axillobifemoral bypass, explantation of the infected aortic graft, and infrarenal aortic ligation. His co-morbidities included ischemic heart disease and hyperlipidemia. The CT angiogram from this admission showed focal thickening of a segment of the duodenum that was tethered to the aortic stump (after previous ligation). A fat stranding was found in the vicinity, but no active contrast extravasation or retroperitoneal hematoma (Figure 1).

**Figure 2:**
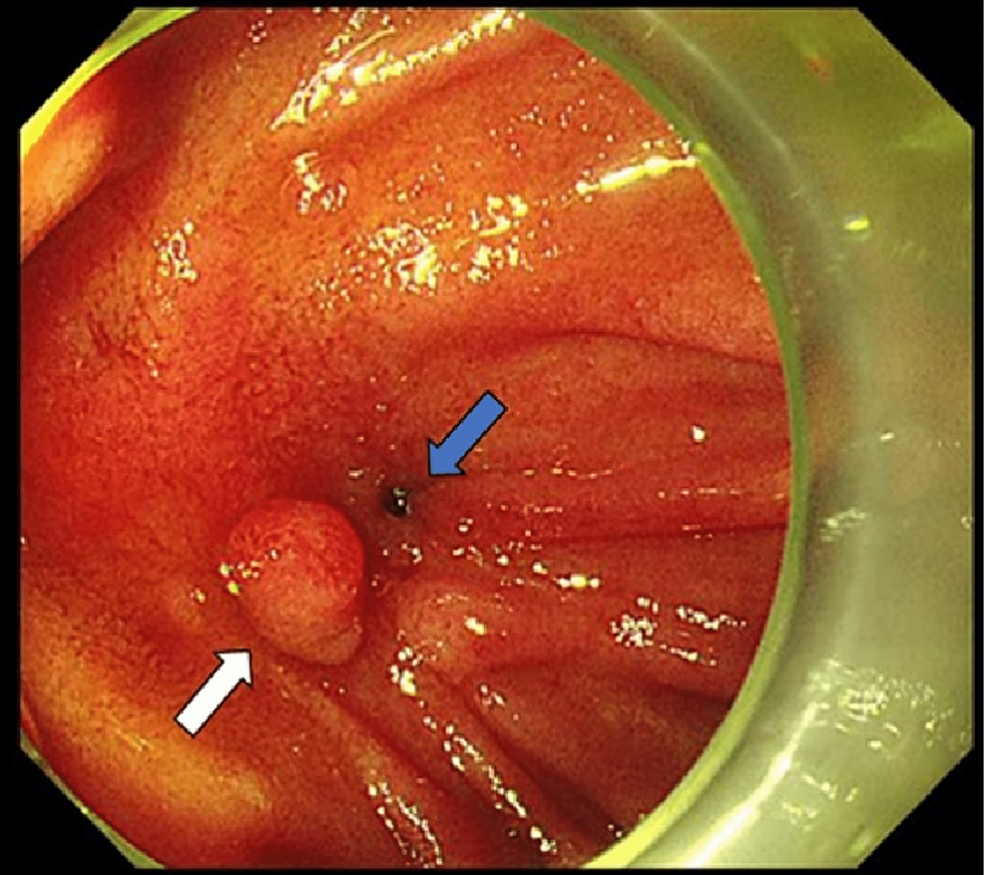
Incidental intestinal polyp in the D3/D4 segment of the duodenum (white arrow) and mucosal defect (blue arrow).

Emergency gastroduodenoscopy demonstrated a pulsating mucosal defect in the posterior wall of the D3/D4 junction (Figure 2).

Endoscopy demonstrated clots but no active bleeding. These findings suggested that the infra-renal aortic stump was bleeding intermittently into the adherent duodenum. Thrombus, inflammatory tissue, and ulcerating bowel mucosa were the only barrier preventing exsanguination into the bowel. The patient was deemed too unsuitable for an emergency open aortic repair in view of his high anesthetic risk from background ischemic heart disease, the current state of hemodynamic shock, the proximity of the defect to the origins of the renal arteries making aortic clamping difficult, and presumed hostile abdomen from multiple prior surgeries. Therefore, we attempted an urgent endovascular approach to manage the crisis. As the patient had a chronically occluded left subclavian artery and prior aortic ligation, there was limited vascular access for an aortic intervention. A 9 Fr arterial sheath (Cook Medical LLC, Bloomington, Indiana) was advanced into the abdominal aorta through a right brachial cut-down. Abdominal aortogram, celiac, and superior mesenteric angiograms were performed. The latter excluded other sources of gastrointestinal bleeding. As this stump was short (around 1 cm), with a tapering geometry, and in close proximity to the renal arteries and superior mesenteric artery (SMA), a novel reconstructive approach had to be adopted.

**Figure 3:**
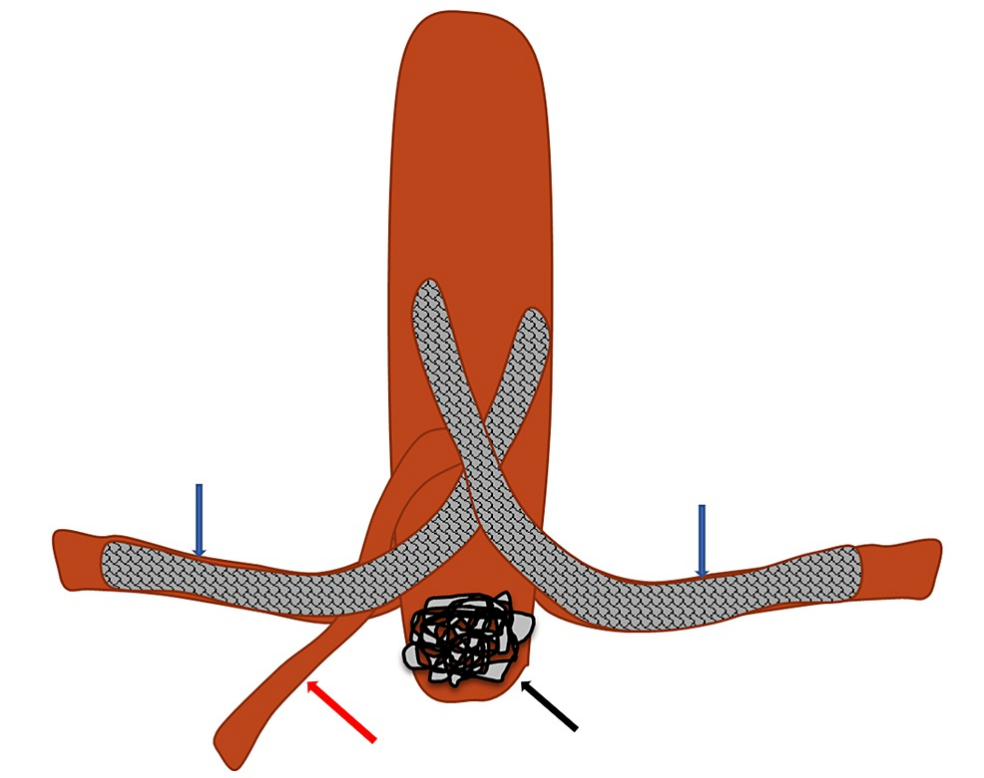
Schematic demonstration of the placement of renal stents in a "chimney-like" configuration (blue arrows), embolic plugs/coils within the aortic sac (black arrow), and relationship to the SMA (red arrow). Illustrated by Dr. Amos Tan. SMA: Superior mesenteric artery.

Both the renal arteries were cannulated, and guidewires were parked simultaneously. A 7 mm x 57 mm Begraft (Bentley InnoMed GmbH, Hechingen, Germany) was advanced into each renal artery in a ‘chimney-like’ configuration, ensuring at least half the length of the stents was in the aorta, with their proximal ends resting on the contralateral aortic wall, to provide them with stability (Figure 3). These were then deployed simultaneously to provide equal luminal flow and prevent the crushing of stents which may be associated with staggered deployment.

**Figure 4:**
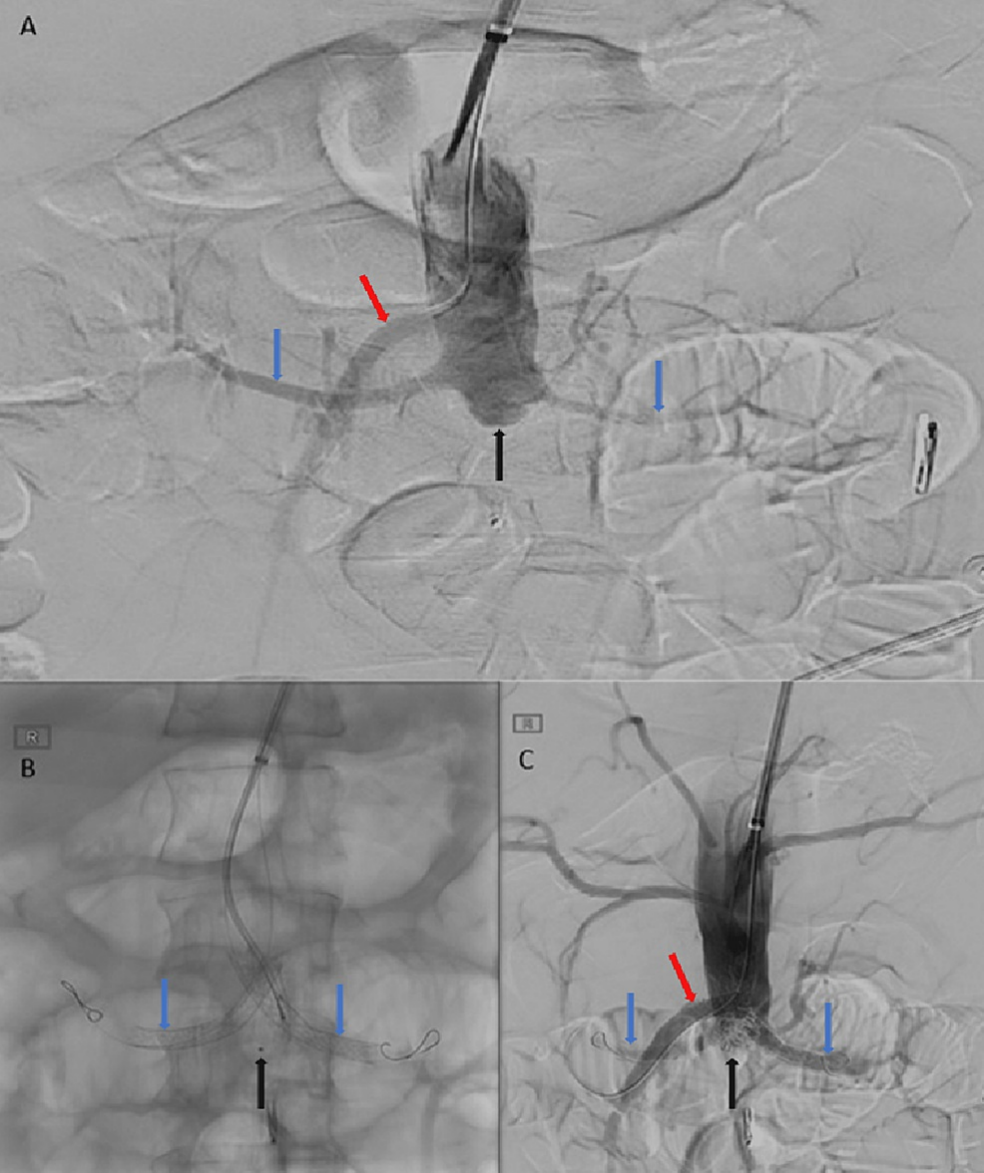
(A) Pre-stenting angiogram demonstrating the relationship of the renal arteries (blue arrows), SMA (red arrow), and aortic sac (black arrow). (B) Angiogram performed after renal artery stenting demonstrates the position of the stents within the renal arteries (blue arrows). A tiny marker within the aortic sac (black arrow) demonstrates the position of the vascular plug. (C) Completion angiogram performed after SMA stenting demonstrates flow within the stented SMA (red arrow) and renal arteries (blue arrows). Vascular plug and coil seen within the aortic sac (black arrow). SMA: Superior mesenteric artery.

A 12 mm Amplatzer 1 vascular plug was deployed into the aortic stump (Abbott Laboratories, Chicago, Illinois). Subsequently, one 4 mm x 350 mm Ruby coil (Penumbra Inc., Alameda, California) was packed into the corners around the plug and below the crossing stents. As the protruding aortic segments of two renal stents could interfere with the flow into the SMA and clot progression from the embolic materials in the aortic stump could encroach the SMA ostium, an 8 mm x 57 mm BeGraft (Bentley InnoMed GmbH, Hechingen, Germany) was deployed into the SMA, once again in a chimney-like configuration. Completion angiogram showed good occlusion of the aortic stump with patent stents (Figure 4).

**Figure 5:**
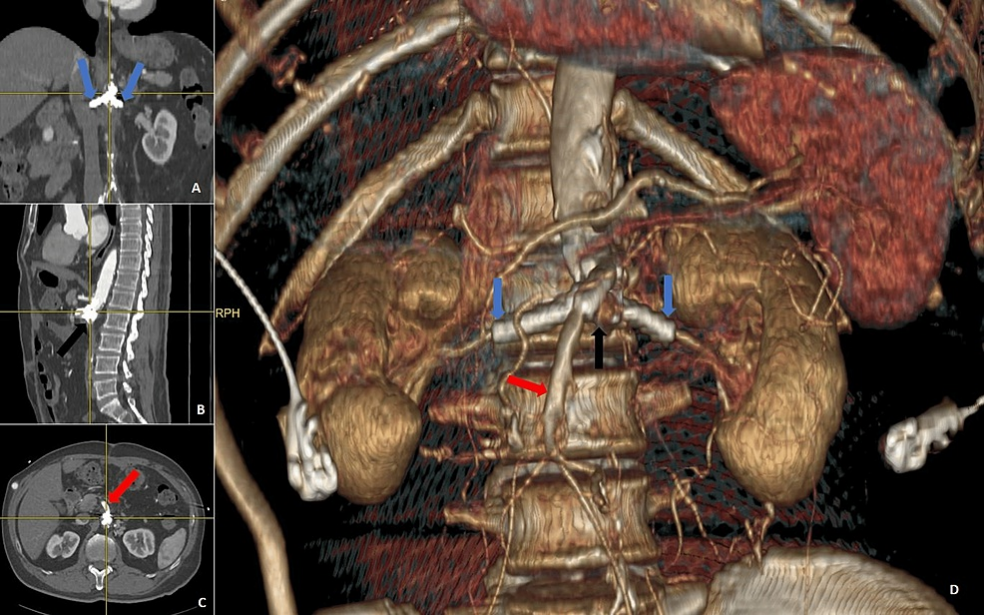
CT aortic angiogram six months post corrective surgery. (A) coronal, (B) sagittal, (C) axial, and (D) 3-dimensional reformat centered on the aortic stump (black arrow) demonstrates good perfusion of the SMA (red arrows) as well as both renal arteries (blue arrows). SMA: Superior mesenteric artery.

Post embolization, there was a stabilization of hemoglobin levels and improvement in inflammatory markers. Eight days later, he underwent an elective surgical exploration. The diseased duodenum, adherent to the aortic stump, was dissected clear of the aortic stump and resected together with a segment of the bowel from D3 to the proximal jejunum. The subsequent duodenojejunal end-to-side anastomosis was performed. The rest of the tissues around the embolized aortic stump was healthy. The patient recovered uneventfully with no recurrent GI bleed. The resected duodenum grew *Enterococcus faecalis* sensitive to amoxicillin. Two weeks later, he was discharged with life-long antimicrobial coverage. CT aortogram performed six months post-surgery demonstrated patency of the superior mesenteric and bilateral renal grafts (Figure 5) with no tethering of bowel loops to the aortic stump, pseudoaneurysm, contrast extravasation or migration of the stents, vascular plug, and coils. The patient remains well at the nine-month follow-up.

## Discussion

SAEF is a rare but lethal entity with limited literature and experience in managing this condition. SAEF is estimated to occur in 0.4-4.0% of patients with prior aortic intervention, which is expected to increase with increased detection of aortic disease and the use of aortic prosthetic grafts [3,4]. The gold standard for the treatment of SAEF is surgical repair which involves graft excision, bowel repair, and extra-anatomic bypass (EAB) or in-situ repair (ISR) either as a staged or single procedure [4]. However, mortality and morbidity of open surgical aortic repair remain high, at 70%, with most complications being GI (30%), pulmonary (25%), and renal (21%) [4]. The rate of recurrence of SAEF after the repair is also estimated at 4% [2]. In the last decade, there has been greater interest in endovascular options, especially for unstable patients [2]. Pooled data analysis has shown lower in-hospital mortality rates with endovascular repair vs. open repair (7.1% vs. 33.9%), with the best mortality and morbidity rates from staged endovascular repair followed by definitive surgical repair of SAEF (4%) [2]. In this patient, we dealt with the scarcely reported problem of late recurrence of a SAEF treated with previous excision and EAB. In such cases, the options are often very limited, especially when patients come in shock. Within six hours of admission, we managed to arrest the bleeding by creating an approximately 1 cm thick layer of embolic material protecting the aortic stump from bleeding into the bowel. Providing an occlusive scaffold preventing further acute hemorrhage through the fistula provided time for us to optimize the patient for general anesthesia and conditions for explorative surgical repair. Interval surgical exploration was vital to remove the adherent bowel loop, a potential source for infecting the aortic stump.

To the best of our knowledge, two similar cases have been reported before. The first case used an endovascular plug as a temporizing measure to effectively stop hemorrhage from an AEF in a patient with chronically atherosclerotic infrarenal aorta [5]. The endovascular plug was subsequently found to be protruding outside the aortic wall during surgical repair and was removed intraoperatively [5]. There was no account of the follow-up of this patient. The second case used embolization coils instead of a vascular plug for a patient with recurrent SAEF [6]. Like in our case, their patient had prior aortobifemoral graft explantation and infrarenal aorta ligation with a resultant short aortic stump. They also used bilateral renal chimney grafts to prevent iatrogenic occlusion of the renal arteries. However, their patient was deemed unfit for subsequent surgical exploration. Eleven months later, the patient presented with massive hematemesis. The embolization coils had migrated caudally through the recurrent AEF; the patient subsequently demised. In view of the risk of vascular plug erosion and migration in the long run, we tried to minimize the radial force acting on the aortic stump. Instead of oversizing the vascular plug as per normal practice for embolization (to prevent migration), we utilized a 12 mm vascular plug for a 12 mm vessel lumen and side filled with very soft packing coils to simulate thrombus formation without significant radial force on the stump wall. Timely diagnosis is key in the management of emergencies like SAEF. CT has a widely variable sensitivity (40%-90%) and specificity (33%-100%) for the diagnosis of AEFs [7]. Direct contrast extravasation from the fistula is rarely seen on CT, and secondary signs such as closely apposed bowel loops and perigraft/periaortic soft-tissue thickening are often non-specific. Hence, correlation with clinical history, signs of "herald bleeds," and endoscopic findings are necessary for an accurate diagnosis [7]. Treatment often needs to be customized and instituted with urgency. Finally, the role of antibiotic therapy is vital to prevent a recurrence. The embolic barricade merely provided a scaffold for healthy tissue to grow and heal. Without infection control, this healing mechanism would have fallen apart, and the aortic stump would have evolved into a mycotic aneurysm, despite the embolization [8]. Given the potential for delayed infection, we have planned for lifelong antimicrobial therapy for this patient.

## Conclusions

Recurrent SAEF after graft excision and EAB are rare but challenging to treat and diagnose. Imaging findings are often inconclusive, and correlation with endoscopic and clinical findings is mostly necessary for diagnosis. Treatment is also often time urgent, and endovascular treatment is essential in the acute setting to stabilize the patient and prevent exsanguination. Hence, we hope that by sharing our considerations and method of our customized approach, we have added to the current understanding of this condition.
